# Intelligence and ambition are distributed equally around the globe

**DOI:** 10.1186/1742-4690-7-67

**Published:** 2010-08-13

**Authors:** Kuan-Teh Jeang

**Affiliations:** 1 The National Institutes of Health, Bethesda, MD, USA

## Abstract

The impact of freely accessible knowledge distribution platforms is briefly discussed.

## 

At a recent international conference, I heard former United States president Bill Clinton speak. As a part of his remarks, Clinton commented that in his visits to many developing countries around the world he has found that "The distribution of intelligence and ambition around the world is equal, but the access to opportunities is not" (my paraphrasing). Clinton's point echoes a message of Malcolm Gladwell's book "Outliers"; both argue that a critical key to success is less about who you are or where you are, but whether you have (not) access to the same opportunities as others.

Open Access publishing is fundamentally about providing opportunities (i.e. knowledge) to all in an equally accessible platform. I have noted previously that this principle, beyond all else, was the prime motivation for founding *Retrovirology *[1]. While the Open Access publishing movement and its egalitarian approach to knowledge dissemination are laudable, one should not neglect to highlight other equally worthy efforts to distribute knowledge freely. One such remarkable project is the MIT OpenCourseWare (OCW) initiative http://ocw.mit.edu) which provides free access via the internet to the academic contents (video lectures, syllabus, problem sets, exam solutions...) of approximately 2,000 courses taught by 33 academic departments. There is an immense demand for OpenCourseWare as evidenced that it is being accessed 1.5 million times each month by nearly 1 million unique visitors [2], many outside of the United States.

The lasting impact of paradigm-changing platforms such as Open Access and MIT OCW remains to be quantified. On an anecdotal level, my impression from editing *Retrovirology *suggests that greater accessibility does translate into more impact. For instance, I examined the most accessed *Retrovirology *papers over the past 12 months that were published in the preceding two years. Amongst the top nine most highly accessed original research articles, there are three papers which were published in either 2008 or 2009 [3-5]. These papers have been accessed over the past year 9675, 4399, and 5909 times, respectively. Each is also in the 10% of highest cited *Retrovirology *papers of a similar vintage. Thus while papers need not be highly read in order to be highly cited (e.g. review articles [6,7] are highly cited without necessarily being highly accessed), a large proportion of well cited papers is made up of those articles that are the most frequently read.

The gratification from editing *Retrovirology *is the realization that Open Access and other similar information distribution initiatives are doing well by doing good.

## References

[B1] Jeang KT The Retrovirology Open Access experience Retrovirology 2009 6 115 10.1186/1742-4690-6-115 20003491 PMC2800104

[B2] d'Oliveira C Carson S James K Lazarus J SPORE series winner. MIT OpenCourseWare: unlocking knowledge, empowering minds Science 2010 329 525 526 10.1126/science.11826962 20671179

[B3] Humbert M Rasmussen RA Song R Ong H Sharma P Chenine AL Kramer VG Siddappa NB Xu W Else JG Novembre FJ Strobert E O'Neil SP Ruprecht RM SHIV-1157i and passaged progeny viruses encoding R5 HIV-1 clade C env cause AIDS in rhesus monkeys Retrovirology 2008 5 94 10.1186/1742-4690-5-94 18928523 PMC2576354

[B4] Andrew AJ Miyagi E Kao S Strebel K The formation of cysteine-linked dimers of BST-2/tetherin is important for inhibition of HIV-1 virus release but not for sensitivity to Vpu Retrovirology 2009 6 80 10.1186/1742-4690-6-80 19737401 PMC2754425

[B5] Hohn O Krause H Barbarotto P Niederstadt L Beimforde N Denner J Miller K Kurth R Bannert N Lack of evidence for xenotropic murine leukemia virus-related virus (XMRV) in German prostate cancer patients Retrovirology 2009 6 92 10.1186/1742-4690-6-92 19835577 PMC2770519

[B6] Goila-Gaur R Strebel K HIV-I Vif, APOBEC, and intrinsic immunity Retrovirology 2008 5 10.1186/1742-4690-5-51 18577210 PMC2443170

[B7] Foster JL Garcia JV HIV-1 Nef: at the crossroads Retrovirology 2008 5 10.1186/1742-4690-5-84 18808677 PMC2563024

